# Longitudinal Assessment of an 800 µg Dose of HEBERSaVax in Non-Human Primates over Six Months

**DOI:** 10.3390/vaccines14030230

**Published:** 2026-02-28

**Authors:** Camila Canaán-Haden, Isabel Gonzalez-Moya, Monica Bequet-Romero, Olivia Cabanillas-Bernal, Rafael Martínez-Castillo, Camilo Cerioli-Pentón, Nayelis Chavez-Castro, Marta Ayala-Ávila, Jorge Castro-Velazco, Alexei F. Licea-Navarro, Yanelys Morera-Díaz

**Affiliations:** 1 Pharmaceuticals Department, Biomedical Direction, Center for Genetic Engineering and Biotechnology (CIGB), Playa Cubanacán, Havana 10600, Cuba; camila.canaanhaden@cigb.edu.cu (C.C.-H.); isabel.gonzalez@cigb.edu.cu (I.G.-M.); monica.bequet@cigb.edu.cu (M.B.-R.); rafael.martinez@cigb.edu.cu (R.M.-C.); camilo.cerioli@cigb.edu.cu (C.C.-P.); nayelis.chavez@cigb.edu.cu (N.C.-C.); marta.ayala@cigb.edu.cu (M.A.-Á.); jorge.castro@cigb.edu.cu (J.C.-V.); 2 Secretary of Science, Humanities, Technology and Innovation (SECIHTI), Innovation and Development Promotion Direction, Centro de Investigación Científica y Educación Superior de Ensenada (CICESE), Ensenada 22860, Mexico; cabanillas@cicese.mx; 3 Biomedical Innovation Department, Centro de Investigación Científica y Educación Superior de Ensenada (CICESE), Ensenada 22860, Mexico; alicea@cicese.edu.mx

**Keywords:** HEBERSaVax, VEGF vaccine, immunogenicity, safety

## Abstract

Background/Objectives: HEBERSaVax is a therapeutic cancer vaccine based on recombinant human VEGF antigen adjuvated with VSSP or Aluminum Phosphate (AP). Clinical trials demonstrated the vaccine’s safety and tolerability, with predominantly mild to moderate (grade 1–2) local adverse events. Initial dose optimization studies using the VSSP (Center of Molecular Immunology (CIM), Havana, Cuba) adjuvant showed that increasing the antigen dose to 800 μg significantly enhanced immunogenicity, as measured by improved seroconversion rates, stronger blockade of VEGF/VEGFR1-2 interactions, and reduced platelet-derived VEGF levels. Methods: The AP adjuvant was used to perform essential preclinical validation in non-human primates to support the transition of the 800 μg antigen dose to Phase II clinical trials (CENTAURO-4 and CENTAURO-6). Results: HEBERSaVax adjuvated with AP induced: (1) robust humoral responses with high-titer anti-VEGF antibodies (peak 1:15,000), (2) functional biological activity, specifically the suppression of VEGF-mediated signal transduction, in 90% (9/10 animals), and (3) measurable cellular immune responses. All immunogenic effects were achieved without evidence of systemic toxicity, confirming the safety profile of this preparation. Conclusions: These findings provide compelling preclinical evidence that the 800 μg HEBERSaVax/AP combination maintains the immunogenic potential previously observed with VSSP while demonstrating an equally favorable safety profile. The results strongly support continued clinical development of this VEGF-targeted immunotherapy for cancer treatment.

## 1. Introduction

The CIGB-247 vaccine (designated as HEBERSaVax) represents a novel immunotherapeutic strategy targeting vascular endothelial growth factor (VEGF)-mediated pathologies [1]. As a recombinant human VEGF-based vaccine, it leverages this key angiogenic factor as both antigen and therapeutic target. VEGF serves as a critical mediator of pathological angiogenesis and vascular permeability, driving disease progression in oncology through tumor neovascularization and immune evasion [2,3]. HEBERSaVax utilizes a dual mechanism to rectify aberrant VEGF signaling by engaging both humoral and cellular immunity to neutralize VEGF and eliminate VEGF-expressing tumor cells.

Preclinical development of HEBERSaVax demonstrated its efficacy in murine models, where vaccination induced robust VEGF-specific antibody production and cytotoxic T-cell responses, leading to significant inhibition of tumor growth and metastatic spread. These effects were observed across multiple dosing regimens and adjuvant combinations, with the most common adjuvants being VSSP (a bacterial-derived immunomodulator) and alum salts [1], each contributing distinct advantages [4,5,6]. Alum adjuvants, in particular, are a cornerstone of human vaccinology due to their favorable safety profile and ability to potently enhance antibody responses, making them an attractive candidate for scalable therapeutic vaccine development [7].

Following successful preclinical studies, CIGB-247 progressed to clinical evaluation through the CENTAURO trial (first-in-human Phase I study), which established the safety and immunogenicity of doses ranging from 50 to 400 µg in combination with VSSP. This trial demonstrated a clear dose–response relationship, showing that higher antigen doses correlated with stronger immune activation, including increased seroconversion rates and enhanced VEGF-neutralizing capacity [8].

Building on these findings, the subsequent CENTAURO-2 (Phase Ib) trial investigated escalated dosing strategies, evaluating adjuvant-antigen combinations containing 200, 400, and 800 µg antigen with VSSP, and 200 and 400 µg antigen with aluminum phosphate (AP) [9]. The study confirmed that increasing the antigen dose further enhanced immunogenicity, as evidenced by: (a) higher rates of anti-VEGF antibody production, (b) improved blockade of VEGF interactions with VEGFR1, VEGFR2, and therapeutic monoclonal antibodies (e.g., bevacizumab), and (c) significant reductions in circulating platelet-derived VEGF levels, indicating systemic biological activity. These results provided a strong rationale for advancing the 800-µg dose into Phase II trials (CENTAURO-4 for ovarian cancer and CENTAURO-6 for hepatocellular carcinoma). However, while the 800-µg dose with VSSP had been well-characterized in clinical trials, its combination with alum required further preclinical validation to ensure comparable immunogenicity and safety.

Although AP is a well-established adjuvant with a favorable safety profile, its effects at high antigen doses (800 µg) in combination with VEGF remained underexplored. Key unresolved questions included: (a) whether AP could induce a Th1-skewed immune response (critical for antitumor efficacy) at this dose level; (b) potential differences in antibody affinity or epitope specificity compared to VSSP-adjuvanted vaccines; and (c) safety implications, particularly regarding off-target effects due to VEGF’s physiological roles in vascular homeostasis. Previous studies in *Chlorocebus aethiops* established the safety of CIGB-247 (HEBERSaVax) at doses up to 400 µg with VSSP adjuvant, demonstrating transient hematological changes but no evidence of treatment-related systemic toxicity based on comprehensive clinical monitoring [10]. However, advancing to Phase II trials (CENTAURO-4/6) required evaluation of an 800-µg dose paired with AP—a clinically scalable adjuvant with a well-documented safety profile but uncharacterized compatibility with high-dose VEGF antigens.

To address these gaps, we conducted a comprehensive preclinical assessment in *Chlorocebus aethiops sabaeus* (cynomolgus monkeys), a non-human primate (NHP) model with 99% VEGF similarity to humans, ensuring translational relevance. Our study mirrored proposed clinical regimens, incorporating longitudinal immune monitoring (antibody titers, neutralizing capacity, T-cell responses), clinical and biochemical safety assessments, and exploratory evaluation of AP as an alternative adjuvant to optimize Th1/Th2 balance. This work not only supports the clinical development of HEBERSaVax but also contributes to the broader field of anti-angiogenic cancer vaccines by delineating adjuvant-specific effects at high antigen loads and establishing safety benchmarks for VEGF-targeted therapies in translational models. Here, we present the first systematic evaluation of the 800 µg HEBERSaVax dose in NHP models, bridging critical gaps between preclinical and clinical development.

## 2. Materials and Methods

### 2.1. Vaccine Antigen and Adjuvant

The study employed CIGB-247 antigen (CIGB, Havana, Cuba), a GMP-produced recombinant fusion protein (Batch D911VE/0, NP: 5261C) consisting of human VEGF121 isoform (mutated at E82A/E84A/E86A to block VEGFR2 binding) fused to *Neisseria meningitidis* P64k protein. The antigen was expressed in *E. coli*, purified through sequential IMAC and Sephadex G25 chromatography at the Development Unit of CIGB (Havana, Cuba), and then lyophilized in 400 μg single-dose vials. For immunization, vials were reconstituted with sterile water for injection and adsorbed onto AP adjuvant (0.70 mg Al^3+^/dose) following standard protocols.

### 2.2. Antigen Adsorption Studies

The adsorption kinetics and binding capacity of CIGB-247 to AP (AlPO_4_; 0.70 mg Al^3+^/dose) were evaluated by incubating increasing antigen concentrations (100–800 μg/mL) with the adjuvant in a 1:1 (*v*/*v*) ratio (1 mL antigen + 1 mL AP) up to 60 min at room temperature, every 10 min. Post-incubation, samples were centrifuged (4000 rpm, 5 min) and filtered through 0.2 µm Durapore filters to separate adsorbed complexes from free antigen. Adsorption efficiency was quantified by loading 100 µL of non-adsorbed antigen in size exclusion chromatography (Superdex 200 increase XK10/30 column at 0.5 mL/min flow of Tris 10 mM pH 7.4 buffer. These experiments assessed AP’s saturation threshold, binding kinetics, and suitability for stabilizing high-dose preparations (e.g., 800 μg) in subsequent in vivo studies.

### 2.3. Animal Model and Ethical Considerations

Twelve young African green monkeys (*Chlorocebus aethiops sabaeus*), weighing 3–7 kg and estimated to be 4–8 years old, were included in the study. All animals were individually housed in purpose-designed cages. The cohort was divided into two groups: a treatment group (*n* = 10; 7 males and 3 females) and a procedural control group (*n* = 2; 1 male and 1 female). All animals were sourced from the National Centre for Animal Breeding (CENPALAB, Havana, Cuba), a facility with documented pathogen-free breeding colonies, and maintained at the Center for Genetic Engineering and Biotechnology (CIGB, Havana, Cuba) under conditions compliant with Cuban national standards (NC/ISO 10993-2:2014) [11] and international guidelines (Directive 2010/63/EU) [12].

Animal care followed the principles of the American Association for the Accreditation of Laboratory Animal Care (AAALAC), strictly adhering to the 3Rs principle (Replacement, Reduction, Refinement). The study protocol (Protocol No. CICUAL/CIGB/21100) received full approval from the CIGB Institutional Animal Care and Use Committee (IACUC Approval No. CICUAL/CIGB/21100), which includes an external ethics consultant. Before experimentation, all subjects underwent a 14-day acclimatization period with daily health monitoring by veterinary staff, enrichment protocols, progressive habituation to handling procedures, ad libitum access to autoclaved water, and a standardized primate diet. CBC/clinical chemistry panels at baseline and contingency plans for humane endpoints (including predefined criteria for euthanasia). This housing and care protocol maintained all animals at <10% baseline weight fluctuation and zero mortality throughout the study period

### 2.4. Animal Husbandry and Feeding Protocol

All animals were maintained on a standardized nutritional regimen consisting of commercial primate chow (ALY 1600 formula, CENPALAB) provided twice daily (100 g/kg body weight in the morning, supplemented with 50 g/kg fresh fruits/vegetables in the afternoon), formulated to meet NRC non-human primate nutritional requirements. Acidified ozonated water (pH 6.5–7.0, 0.1–0.2 ppm ozone) was provided ad libitum in sanitized 200 mL polycarbonate bottles, refreshed twice daily. Dietary intake was monitored and adjusted to maintain stable body weights (±5% baseline), with all feeding equipment sterilized daily by autoclaving (121 °C, 20 min). This protocol ensured consistent nutritional status while minimizing gastrointestinal pathogens through water ozonation and food sterilization.

### 2.5. Experimental Groups and Treatment Protocol

A longitudinal, within-subjects design was employed to evaluate immunogenicity, with primary endpoints defined as the changes from pre-immunization baseline in the vaccinated cohort. Twelve *Chlorocebus aethiops sabaeus* monkeys were allocated into two groups: a treatment group (*n* = 10) receiving 800 µg of CIGB-247 antigen adsorbed to 0.70 mg Al^3+^ AP adjuvant in a 1 mL formulation, and a small procedural control group (*n* = 2) administered an adjuvant-only placebo (0.70 mg Al^3+^). The 5:1 group ratio prioritized statistical power for evaluating vaccine responses, while maintaining sufficient controls to monitor for effects attributable to the environment, injection procedure, and adjuvant platform. Individual pre-immunization values served as baselines for each animal. All animals received identical subcutaneous immunization schedules with alternating interscapular injection sites. Animals were uniquely identified through ISO-compliant labels and color-coded cage cards containing study IDs, group assignments, and vaccination timelines. The protocol included daily health monitoring, scheduled weight/temperature checks, and serum collections to assess safety and immunogenicity endpoints, following OECD 407 guidelines for biological evaluation. Random numbers were generated using the Aleator Software “2N” (Version 1.2) computer tool developed at the University of Arkansas, USA.

### 2.6. Immunization Protocol

Animals received subcutaneous injections of either 1 mL of the vaccine (800 μg CIGB-247 antigen adsorbed to 0.70 mg Al^3+^ as AP adjuvant) or placebo (adjuvant-only) in the interscapular region, with alternating injection sites between administrations. The immunization schedule consisted of a primary phase (four bi-weekly doses on Days 0, 14, 28, and 42) followed by monthly boosters through Week 24. All procedures were performed under ketamine sedation (5–10 mg/kg IM) using sterile preparations verified to have >75% antigen adsorption by size exclusion chromatographic evaluation of free antigen in vaccine preparation (Supplementary Figure S1). Animals were monitored for local reactions (erythema, swelling at 1 h, 24 h, and 72 h post-injection) and systemic effects (daily clinical assessments for 7 days post-immunization), with serum collected bi-weekly during priming and monthly during maintenance phases for immunogenicity analysis. The protocol complied with 3Rs principles, incorporating minimized group sizes, refined anesthesia protocols, and comprehensive safety monitoring to assess acute immune responses and long-term safety profiles.

### 2.7. Clinical Evaluations and Monitoring

All study animals underwent comprehensive clinical monitoring throughout the trial period, beginning with daily behavioral assessments (food/water intake, locomotor activity, and neurological status) and systematic physical examinations of mucosal membranes, lymph nodes, and integumentary, respiratory, digestive, and nervous systems. Quantitative measurements included body weight (days 0, 14, 28, 42, 49, 56, 70, 77, 98, 120, 140, and 174) using calibrated digital scales (±0.1 kg) and rectal temperature measurements on the same schedule with veterinary-grade thermometers. Blood samples collected on days 0, 59, and 174 were processed under standardized conditions (30 min clot formation at room temperature, centrifugation at 10,000× *g* for 10 min at 4 °C) before serum analysis within 72 h using a Cobas Integra 400 PLUS analyzer (Roche Diagnostics, Basel, Switzerland) for hematological parameters, biochemical markers (ALT, AST, creatinine, bilirubin), immunoglobulin levels, and 12 additional metabolic indicators. A dedicated six-month neurological sub-study concurrently evaluated chronic effects through behavioral, autonomic, and motor function assessments. All clinical data were benchmarked against institutional historical controls from healthy primates, with continuous veterinary oversight to monitor potential adverse effects during the study. Due to blood volume constraints, sample collection from the small procedural control group was prioritized for baseline and select intermediate time points to monitor for gross adverse effects. At the same time, comprehensive longitudinal biochemistry was performed on the vaccinated cohort.

### 2.8. Immunological Evaluations

#### 2.8.1. ELISA Reagents and Procedures

Skim milk powder (Cat# 1.15363.0500) and TMB (Cat# 613544) were supplied by Merck (Darmstadt, Germany). Sigma provided phosphate-buffered saline (PBS) (Cat# D1408). Tween 20 solution (Cat# A1389) was supplied by AppliChem (Darmstadt, Germany), along with in-house produced GST-hVEGF121 [13]. HRP-conjugated goat anti-human IgG polyclonal antibody (Jackson Immunoresearch Laboratories, Cat# 109-035-098, West Grove, PA, USA) was used at 80 ng/mL for detecting human serum IgG. Bevacizumab (Roche, West Grove, PA, USA) served as the positive control for the functional bioassay. VEGF165 (Cat# J2371, Promega, Madison, WI, USA) was used as the signal-activating molecule *in vitro*.

#### 2.8.2. Anti-VEGF Antibody Quantification

Serum IgG titers against VEGF were determined by ELISA using GST-hVEGF121-coated plates (Costar, 3590, New York, NY, USA), following established protocols [14]. Titers were calculated from linear regression curves of serial dilutions, with the threshold as five times the standard deviation above pre-immune serum averages.

#### 2.8.3. Functional Bioassay

The functional blockade of VEGF signaling was evaluated using a HEK293 reporter cell system (KDR/NFAT-RE) [15]. Cells were seeded at 40,000 per well and incubated with test sera diluted 1:24 in the presence of VEGF165 (33.3 ng/mL) for 6 h. Luciferase activity was then detected using the Firefly luciferase filter (580 ± 20 nm) and Bio-Glo substrate (Cat# G720A, Promega, Madison, WI, USA). These specific assay conditions were selected based on manufacturer recommendations and on preliminary studies of assay establishment. The percentage of inhibition was calculated from luminescence values using the formula: 100%—(sample RLU/maximum RLU × 100). Assay parameters were established using bevacizumab (0.66 µg/mL) as a positive control and medium-only wells as a background control.

#### 2.8.4. Direct Cell Cytotoxicity Assay with Monkey Peripheral Blood Mononuclear Cells (PBMC)

For this evaluation, the five animals exhibiting the highest antibody titers following immunization were selected. Since the alum adjuvant used in the formulation preferentially induces a Th2-type immune response (characterized by strong antibody production), this assay aimed to assess whether a cellular cytotoxic response could be elicited qualitatively.

Samples of heparinized blood were collected from these selected monkeys seven days after the final booster. Peripheral blood mononuclear cells (PBMC) were isolated using a Ficoll gradient and maintained at a density of 1 × 10^6^ cells/mL in RPMI-1640 medium supplemented with 10% fetal bovine serum (FBS) and phytohemagglutinin (PHA). For the assay, PBMC from each animal served as target cells. A portion of these target cells was incubated with the immunization antigen for 2 h, while another portion remained untreated. All target cells were subsequently labeled with carboxyfluorescein succinimidyl ester (CFSE, Cat# 21888, Sigma, St. Louis, MO, USA) as previously described [16]. The labeled target cells were then mixed with autologous PBMC (effector cells) at a 2:1 effector-to-target ratio. Direct cytolysis was evaluated by flow cytometry and expressed as the percentage reduction in the CFSE-labeled target cell population [17].

### 2.9. Statistical Analysis

All statistical analyses were performed using GraphPad Prism version 10.1 (GraphPad Software, San Diego, CA, USA). The normality of data distribution was assessed using the Shapiro–Wilk test. As the study was not powered for formal statistical comparisons between the vaccinated and procedural control groups, the primary analysis focused on changes from baseline levels within the immunized group (*n* = 10). Comparisons between individual post-immunization time points and the pre-immunization baseline were made using paired statistical tests: the paired *t*-test for normally distributed data or the Wilcoxon signed-rank test for non-normally distributed data. For longitudinal data, one-way repeated-measures ANOVA was used. The robustness of ANOVA to minor deviations from normality was considered. Post hoc analyses consisted of planned comparisons. Timepoints were compared to the pre-immunization baseline using Dunnett’s correction, which was applied in its form adapted for repeated-measures designs (effectively applying Dunnett’s logic and distribution to the standard errors and degrees of freedom from the repeated-measures model), as correctly implemented in the statistical software. This preserved the family-wise error rate for this specific family of comparisons. Data are presented as either mean ± standard deviation (SD) for descriptive statistics as specified in figure legends. All tests were two-tailed with statistical significance defined as *p* < 0.05. Sample size justification was based on power analysis (β = 0.8, α = 0.05) using preliminary data from pilot studies.

## 3. Results

### 3.1. Humoral and Cellular Immune Response in PNH Immunized with HEBERSaVax

**Figure 1:**
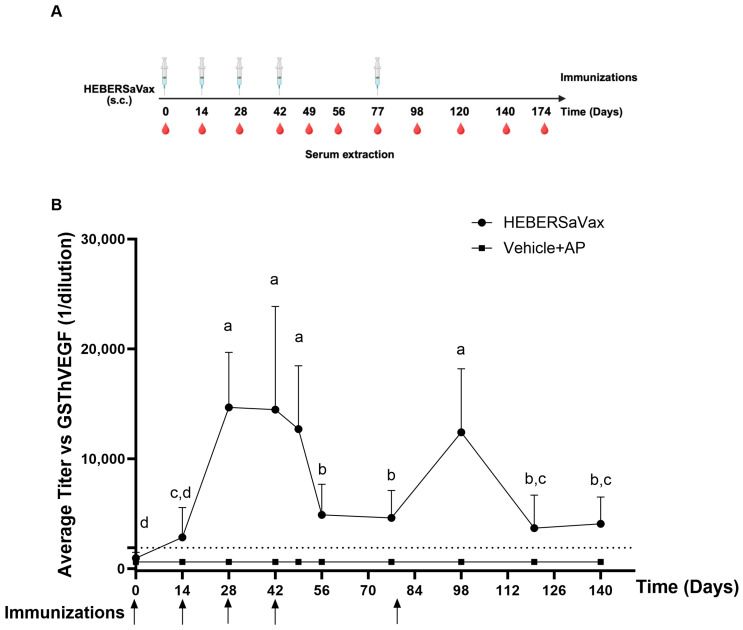
Vaccine-induced humoral immunity in non-human primates. (**A**) Immunization protocol: Cynomolgus monkeys (*n* = 10/group) received four subcutaneous doses of 800 μg HEBERSaVax (CIGB-247 + AP adjuvant) on days 0, 14, 28, 42, and 77 (syringes). (**B**) Anti-VEGF antibody kinetics: Mean ± SD against human VEGF121 measured by quantitative ELISA. Each point represents the mean of duplicate measurements from individual animals. Statistical significance for the vaccinated group was determined by a one-way repeated-measures ANOVA with Dunnett’s multiple-comparisons test versus the pre-immunization (Day 0) baseline. Different letters indicate statistical significance. Arrows indicate the immunization days. The dashed line indicates the seropositivity threshold (3× pre-immune levels).

The animals were immunized with 800 µg of the antigen or the vehicle in combination with 0.7 mg of AP on bi-weekly schedules (Figure 1A). The 800 µg aluminum phosphate-adjuvanted CIGB-247 combination elicited robust anti-VEGF antibody responses in all immunized animals (*n* = 10), demonstrating classic T-cell dependent immunization kinetics (Figure 1B). Analysis by one-way repeated measures ANOVA with Dunnett’s multiple comparisons test identified three distinct phases: (1) An initial prime-boost phase where 40% of animals (4/10) achieved titers >1:1900 (3× pre-immune levels cut off) by Day 14, progressing to 100% seroconversion by Day 28 with peak titers (mean 15,000 ± 3200; 15-fold increase over baseline, *p* < 0.0001), which were effectively maintained through Days 42–49 (12,000–14,500; *p* = 0.12 between timepoints) following third and fourth immunizations; (2) A rest phase showing expected physiological decline (55% reduction from Days 49–56: 12,701 ± 5751 to 4885 ± 2792, *p* = 0.0002) followed by stabilization (Days 56–77: 4621 ± 2491, *p* > 0.9999); and (3) A memory recall phase where the Day 77 booster rapidly restored titers (12,416 ± 5761, *p* = 0.0008 vs. Day 77 baseline), with subsequent decline to 3683 ± 3011 (*p* = 0.003 vs. peak) and stabilization. This kinetic confirm the capacity of the vaccine to establish durable humoral immunity while remaining responsive to antigen re-challenge—a critical feature for achieving sustained VEGF suppression in cancer therapy. Additionally, the individual anti-VEGF antibody kinetics for all study animals are shown in Supplementary Figure S2. These individual data confirm a consistent and sustained response in each subject, which aligns with the mean and standard deviation of the entire cohort presented in Figure 1B.

**Figure 2:**
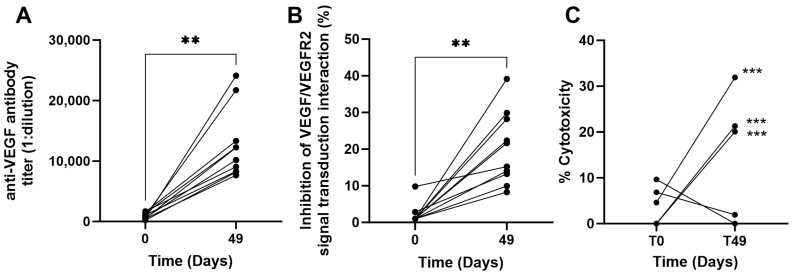
Humoral and cellular immune response in PNH immunized with HEBERSaVax. (**A**) Anti-VEGF antibody titers against human VEGF_121_ were measured by quantitative ELISA in serum from the same animal cohort at days 0 and 49 of the protocol. (**B**) Inhibition of the signal transduction mediated by the VEGF/VEGFR2 axis with 1:24 dilution of the same serum samples, determined with a VEGF reporter gene assay. (**C**) Direct cytolysis of autologous VEGF-charged PBMC coming from monkeys immunized with HEBERSaVax. Evaluated by FACS as the percent reduction in the CSFE-labeled cell population compared to non-loaded autologous PBMC. Each symbol represents the media of two replicated values. Wilcoxon test, ** *p* ˂ 0.01, *** *p* < 0.001.

The functional capacity of HEBERSaVax-induced antibodies to disrupt VEGF signaling was assessed by a VEGF reporter gene assay (VEGF Bioassay, Promega, Madison, WI, USA). Immunization with 800 µg CIGB-247 adjuvated with AP generated potent VEGF-neutralizing antibodies in 90 % of NHPs (9/10 animals). Quantitative analysis revealed significant enhancement of receptor blockade, with inhibition increasing from baseline levels to day 49 (one week post-fourth immunization; *p* = 0.0020, Wilcoxon paired test) (Figure 2B). The ability to inhibit signal transduction demonstrates that the vaccine elicits antibodies (Figure 2A) capable of effectively interfering with a critical molecular interaction in VEGF-mediated signaling pathways. This result confirms that vaccine-induced antibodies bind VEGF and effectively disrupt its biological activity.

Vaccine-induced cellular immunity was assessed in 50% of animals (5/10 cynomolgus macaques) through direct cytotoxicity assays using CFSE-labeled PBMCs incubated with CHO cells expressing recombinant human VEGF (CHO-rhVEGF). In the HEBERSaVax-immunized group, 60% of animals (3/5 evaluable) developed significant VEGF-specific cytotoxic activity by Day 49 (one week post-fourth immunization), demonstrating 20–32% specific lysis against CHO-rhVEGF targets (mean 26.4% ± 4.1) compared to pre-vaccination baseline levels (8.2% ± 2.3; *p* < 0.01 by paired *t*-test) (Figure 2C).

### 3.2. Safety and Clinical Evaluations

Comprehensive clinical monitoring demonstrated that immunization with 800 μg CIGB-247/AP was well-tolerated, with no treatment-related adverse effects on behavior, neurological function, or systemic health. Daily observations confirmed regular feeding, grooming, and social interactions, while physical examinations revealed no abnormalities in respiratory, digestive, integumentary, or nervous systems. Transient injection-site indurations (5–10 mm), consistent with aluminum adjuvant effects, resolved within 72 h.

**Figure 3:**
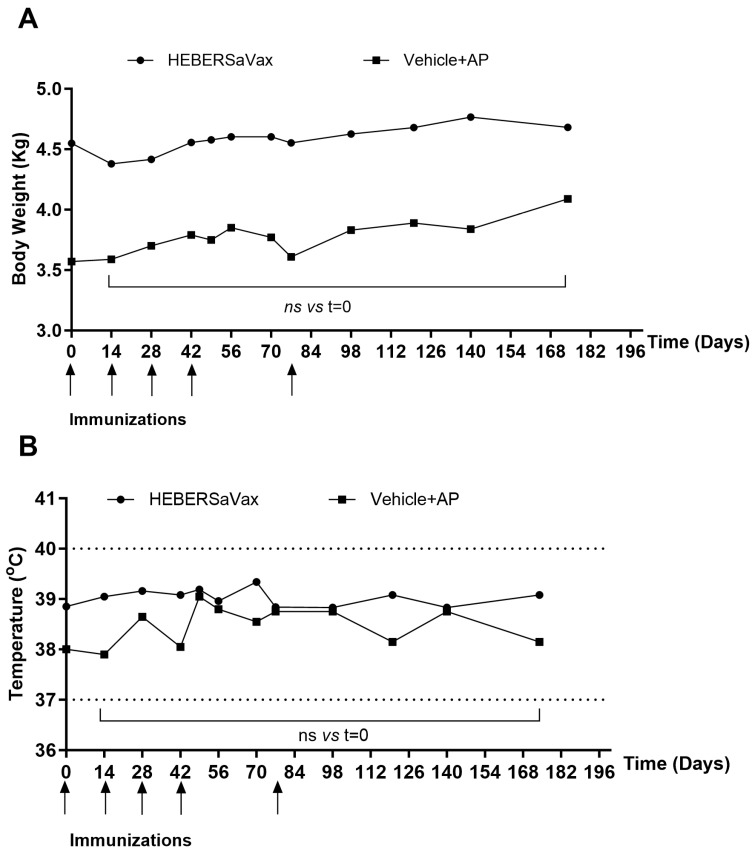
Longitudinal monitoring of physiological parameters in non-human primates (NHPs) immunized with HEBERSaVax (*n* = 10) or Vehicle + AP (*n* = 2). (**A**) Body weight progression (kg) and (**B**) core temperature (°C) measurements throughout the immunization schedule. Data points represent mean values for vaccinated NHPs at each time point. Arrows indicate the immunization days. Dot lines indicate the reported normal physiological range for *Chlorocebus aethiops sabaeus*. Statistical analysis for the immunized group with HEBERSaVax was performed using a one-way repeated-measures ANOVA with Dunnett’s multiple-comparisons test versus the pre-immunization (Day 0) baseline.

Longitudinal tracking of body weight in all animals showed stable physiological growth trajectories consistent with species-specific norms, with no clinically relevant deviations observed in either vaccinated or procedural control subjects. In the vaccinated cohort, body mass progression remained stable over time (mean Δ = 4.582 ± 0.1074 kg), aligning closely with expected growth patterns for the species and showing no adverse trends indicative of systemic or metabolic disruption. Figure 3A illustrates this stable trajectory within the normal physiological range for *Chlorocebus aethiops sabaeus*. The absence of treatment-related shifts in body weight provides substantial evidence supporting the safety profile of the combination, particularly regarding potential metabolic or systemic impacts of chronic VEGF inhibition.

Core temperature profiles (37–40 °C) remained within the species-typical range throughout the study (Figure 3B). All animals, including both vaccinated and procedural control subjects, maintained biochemical markers (ASAT, bilirubin) within normal limits following baseline, with longitudinal profiles showing no clinically meaningful variations. Transient, non-progressive fluctuations in some biochemical parameters (e.g., creatinine) were observed (Table 1). These were not sustained at the study endpoint and, in the absence of correlative clinical findings, were not considered indicative of treatment-related organ dysfunction. Neurological scoring (motor activity, CNS function) detected no abnormalities in any animal, confirming the absence of thrombotic, hemorrhagic, or organ-specific pathologies.

## 4. Discussion

This investigation presents the first comprehensive preclinical assessment of the 800 µg dose of antigen CIGB-247 adjuvated with AP adjuvant in non-human primates, demonstrating robust immunogenicity and a favorable safety profile. Our findings effectively bridge critical knowledge gaps between previous clinical observations and the proposed transition to AP for Phase II-III clinical trials.

HEBERSaVax exhibits fundamentally distinct mechanisms from synthetic anti-angiogenic drugs or monoclonal antibodies like bevacizumab. As an active immunotherapy, it induces: (a) VEGF-blocking antibodies that disrupt endothelial cell signaling through metronomic inhibition of VEGF/VEGFR interactions, and (b) cytotoxic T cells (CTLs) capable of directly eliminating VEGF-producing tumor and stromal cells. The chronic vaccination regimen leverages the properties of the adjuvant to potentially drive tumor epitope spreading while maintaining physiological immune regulation, a critical advantage for this self-antigen vaccine, as demonstrated in analogous platforms [18]. Unlike passive antibody therapies requiring high-dose infusions [19,20,21], HEBERSaVax sustains moderate, controlled levels of both humoral and cellular effectors, creating a sustained anti-tumor response. This dual mechanism, combining angiogenic blockade with direct tumor killing, represents a paradigm shift in VEGF-targeted therapy by addressing both vascular and immunosuppressive aspects of the tumor microenvironment [1].

The immunogenic properties of HEBERSaVax with AP adjuvant were previously characterized across multiple antigen doses and administration regimens. In murine models (C57BL/6 and BALB/c strains), biweekly administration of 100–400 µg doses consistently induced anti-VEGF IgG antibody production, with the 400 µg dose demonstrating optimal immunogenic performance. This dosage regimen generated antibody titers capable of significantly inhibiting VEGF/VEGFR2 interaction assays, while predominantly eliciting an IgG1 subclass profile indicative of a Th2-biased humoral response. The translational significance of these findings was further substantiated in non-human primates, where biweekly administration of 200–400 µg doses exhibited clear dose-dependent enhancement of antibody titers, with the higher dose demonstrating superior immunogenic performance [22]. A particularly noteworthy aspect of this investigation was the comparative evaluation of AP versus VSSP adjuvant systems. While VSSP preparations consistently demonstrated more robust Th1-type responses [23,24], AP offered several distinct advantages: (a) superior antibody titers in specific experimental conditions, (b) comparable therapeutic efficacy with biweekly versus weekly administration schedules (suggesting potential dose-sparing benefits) [22], (c) enhanced regulatory acceptability as an FDA-approved adjuvant [25,26], and (d) more favorable pharmacoeconomic characteristics for clinical development and potential commercialization.

The CENTAURO clinical trial series also provided critical insights into dose–response relationships and adjuvant effects in human subjects [8,9]. These studies demonstrated that both VSSP-adjuvanted (400–800 µg doses) and AP-adjuvanted preparations (200–400 µg doses) maintained acceptable safety profiles while eliciting measurable immunogenicity in cancer patients [9]. Notably, the trial design did not include evaluation of the 800 µg dose with AP, based on preclinical concerns that AP might disproportionately enhance humoral responses beyond therapeutic thresholds. However, subsequent clinical observations revealed that the differential immunogenicity between adjuvants was less pronounced in humans than predicted, with dose escalation (particularly to 800 µg with VSSP) proving to be the primary determinant of enhanced immune responses [9]. This clinical experience naturally prompted consideration of evaluating the 800 µg dose with AP in subsequent trials. However, this transition required resolution of two key preclinical questions: (1) whether the immune response profile would remain balanced at this higher dose with AP, and (2) whether enhanced VEGF inhibition might compromise physiological vascular functions.

Our comprehensive preclinical evaluation conclusively establishes the 800 µg CIGB-247 adjuvated with AP as a scientifically valid candidate for advanced clinical development. The immunogenic characteristics of the 800 µg dose regimen in non-human primates demonstrate significant therapeutic relevance, with the observed seroconversion (40% by Day 14) and attainment of high peak antibody titers (1:15,000) by Day 28 indicating that this combination reaches clinically meaningful thresholds while maintaining optimal pharmacokinetic properties.

The kinetic profile of HEBERSaVax, characterized by rapid seroconversion followed by sustained antibody maintenance through scheduled boosters, provides evidence for robust engagement of both primary and secondary immune pathways [27,28]. This profile is particularly significant for oncology, where rapidly achieving therapeutic antibody levels may be critical for patients with advanced malignancies [29]. The data indicate that the 800 µg dose optimally balances antigenic stimulation and adjuvant activity to facilitate this accelerated response, while also effectively breaking tolerance to self-antigens and promoting B cell activation, dendritic cell presentation, and T follicular helper cell activity [30]. The predictable antibody reduction after treatment cessation and the prompt anamnestic response upon re-immunization confirm the establishment of persistent immunological memory, supporting flexible clinical protocols with an intensive priming phase and periodic maintenance boosters [31]. These collective properties, accelerated seroconversion, durable memory, and recall capacity, address key therapeutic vaccine challenges by reducing the vulnerable treatment-initiation period and enabling adaptable, long-term disease management [32].

To functionally validate the vaccine-induced immune response, we employed a KDR/NFAT reporter gene assay, which directly quantifies inhibition of VEGF-mediated signal transduction. This assay utilizes engineered HEK293 cells stably expressing VEGFR2 (KDR) and an NFAT-driven luciferase reporter, enabling the real-time measurement of pro-angiogenic pathway activation via luminescence [33]. Unlike binding assays (e.g., ELISA), this system provides a physiologically relevant readout of the entire downstream signaling cascade triggered by VEGF-VEGFR2 interaction.

Serum from vaccinated animals potently inhibited VEGF-driven signaling in this assay. The results demonstrate that HEBERSaVax-induced antibodies effectively block ligand-receptor binding and subsequent signal transduction, with the degree of inhibition correlating positively with anti-VEGF antibody titer. This establishes a direct link between the quantitative humoral response and a critical qualitative outcome: the functional disruption of a key angiogenic pathway. These data confirm that immunization generates biologically active antibodies capable of suppressing pro-angiogenic signaling, a cornerstone of the vaccine’s proposed therapeutic mechanism.

This functional analysis was conducted at Day 49, corresponding to peak antibody titers following the primary immunization series. While this time point robustly confirms high bioactivity, the technical demands of the cell-based assay precluded a complete longitudinal functional profile. Consequently, the durability of this neutralization capacity through later memory phases (e.g., post-booster) remains an open question. Future studies featuring serial functional assessments will be essential to determine if neutralization quality is maintained over time in concert with antibody persistence.

A detailed analysis of antibody subclasses (e.g., IgG1/IgG2 ratio) was not performed in this primate study. While we comprehensively characterized the humoral response in terms of titer, kinetics, and function, this omission precludes a direct serological assessment of the Th1/Th2 polarization induced by the 800 µg AP formulation. Foundational work in murine models underscores the importance of such analysis, demonstrating that adjuvant choice critically shapes the IgG subclass profile. For instance, immunization with the VEGF antigen, while eliciting a predominant Th2-biased (IgG1) response, can also induce measurable levels of IgG2a and IgG2b subclasses with specific formulations, a profile suggestive of a mixed Th1/Th2 response [34]. Therefore, future studies incorporating subclass profiling in primate models are warranted to provide deeper insight into the qualitative nature of the immune response elicited by this specific high-dose AP formulation and to inform adjuvant selection for clinical optimization better.

The overall immune response profile was further characterized by evaluating cellular immunity. Notably, 60% of evaluable animals (3 out of 5) developed significant VEGF-specific cytotoxic T-cell (CTL) activity. This indicates that the AP-adjuvanted formulation can engage cellular immunity, a complementary mechanism that may enhance long-term anti-tumor efficacy by targeting VEGF-producing cells. The observed heterogeneity is not uncommon in therapeutic vaccination and may be influenced by individual immunological backgrounds or the sensitivity of the ex vivo assay. Importantly, this cellular response complements the robust and consistent humoral response observed across all animals. This multimodal approach, combining metronomic antibody-mediated inhibition with the potential for direct CTL-mediated killing of VEGF-expressing cells, addresses both angiogenic and immunosuppressive aspects of the tumor microenvironment.

Interestingly, the detection of CTL activity suggests that AP maintains a measurable, though modest, capacity to elicit Th1-polarized immune reactions. The Th1-inducing potential of aluminum salts, while substantially less pronounced than the strong Th1 bias typically associated with VSSP adjuvants, remains biologically relevant.

Contemporary immunological research reveals that aluminum-based adjuvants possess a previously underappreciated capacity to induce and sustain antigen-specific T-cell responses, extending beyond their well-established role in promoting humoral immunity [35]. Notably, when formulated with particulate or structurally optimized antigens, aluminum adjuvants can significantly enhance dendritic cell (DC) activation, leading to increased IFN-γ secretion and a more robust cellular immune profile [36]. Mechanistically, this is driven by the adjuvant’s ability to activate caspase-1-dependent release of IL-1β and IL-18 from DCs, cytokines that support Th1 cell differentiation and function and contribute to IFN-γ production [37]. These cellular mechanisms have been experimentally validated to potentiate anti-tumor immunity, enhancing cytotoxic T-cell activity and tumor control in preclinical models’ responses [33,34]. Although aluminum adjuvants typically skew responses toward antibody production (Th2 bias), their demonstrable induction of a partial but significant Th1 response indicates a broader immunomodulatory potential. In the context of cancer vaccines, this dual capability to stimulate both the humoral and cellular arms of adaptive immunity suggests that aluminum-adjuvanted platforms can contribute to anti-tumor efficacy, particularly when designed to maximize antigen presentation and cross-priming.

The therapeutic targeting of VEGF, while clinically validated in cancer treatment, requires rigorous safety evaluation when high antigen doses (800 µg) are used in active immunotherapy. VEGF’s pleiotropic roles in vascular homeostasis raise legitimate concerns about therapy-induced disruption of critical physiological processes [38,39]. The present investigation addresses these concerns through a comprehensive preclinical assessment of the 800-µg dose, as this elevated antigen load could theoretically amplify both therapeutic and adverse effects. Potential impacts on cardiovascular, hepatic, and renal systems are especially relevant given VEGF’s crucial regulatory functions in these tissues [40,41].

The longitudinal safety profile observed with the 800 µg dose provides critical reassurance for clinical translation. There was no systemic toxicity, no weight loss, no neurological deficits, and no hematological abnormalities, supporting the favorable safety profile of aluminum phosphate even at this high antigen dose. Transient injection-site reactions, typically resolving within 72 h, were consistent with the known local effects of aluminum-based adjuvants [42,43]. Notably, no biochemical or clinical evidence of VEGF-related pathological sequelae emerged. Stable serum parameters further confirm the safety profile of this vaccine formulation for clinical application. This study did not assess specific coagulation parameters (PT, aPTT, D-dimer) [44], which are of translational relevance for chronic VEGF-targeted therapies. Future studies will incorporate these endpoints to characterize the hemostatic safety profile better.

This safety assessment was based on intensive longitudinal monitoring of clinical signs, hematology, and comprehensive serum biochemistry (including renal and hepatic panels) over six months. Although this study did not include histopathological examination of angiogenesis-sensitive organs (e.g., kidney, retina) [45] due to its non-terminal design, the monitoring protocol was explicitly designed to detect systemic consequences of VEGF inhibition. The absence of clinical, behavioral, or biochemical signs indicative of compromised vascular homeostasis, such as unexplained weight loss, hypertension, or markers of organ dysfunction, suggests that chronic active immunization at this dose does not elicit the off-target vascular pathologies associated with high-dose passive VEGF blockade. This multifaceted safety profile aligns with previous toxicological assessments at lower doses and strongly [10] supports the translational safety of this regimen.

Nevertheless, certain study limitations should be noted. Confirmatory histopathological or imaging data from major organs were not obtained, and future preclinical studies with terminal endpoints would benefit from such analyses. However, the primary safety conclusions are robustly supported by the complete longitudinal profile of the vaccinated animals.

## 5. Conclusions

This preclinical study evaluated the fixed, high-dose formulation of HEBERSaVax containing 800 µg of recombinant human VEGF adjuvanted with AP. This specific combination represents an optimized, synergistic formulation, selected based on foundational evidence that the VEGF antigen alone fails to break immune tolerance and that immunogenicity scales with the antigen dose when delivered with an adjuvant. The present work robustly confirms this rationale in a translational non-human primate model, demonstrating that the 800 µg + AP formulation induces potent and durable VEGF-specific immunity characterized by high-titer functional antibodies and a favorable safety profile over six months. By validating the immunogenic and safety profile of this AP-adjuvanted high dose, these findings directly address a key translational gap and complement recent clinical data with other adjuvants. This work confirms the viability of a scalable, well-characterized adjuvant platform and provides the critical foundation to advance this specific formulation into controlled Phase II clinical trials, enabling the strategic clinical evaluation of a promising therapeutic cancer vaccine candidate.

## 6. Patents

Bequet Romero, M., Morera Díaz, Y., Ayala Ávila, M., Gavilondo Cowley, J. V., Sánchez Ramírez, J., Hernández Bernal, F., Gonzalez Blanco, S., Espinosa Rodríguez, L. A., Besada Pérez, V. A., Pérez de la Iglesia, M., Trimino Lorenzo, L., Limonta Fernández, M., & Ubieta Gómez, R. (1 July 2021). Polypeptides comprising mutated forms of human VEGF-A with rearrangements of disulfide bonds and compositions containing the same (World Intellectual Property Organization Patent No. WO2021129898).

## Figures and Tables

**Table 1:** Longitudinal analysis of hematological and serum biochemical parameters in non-human primates immunized with 800 μg CIGB-247 antigen plus AP adjuvant.

Parameter	Physiological Range (Unit)	Treatment	Day 0 (Pre-Vaccination) (Mean ± SD)	Day 56 (Post-Vaccination) (Mean ± SD)	Day 174 (Post-Vaccination) (Mean ± SD)
Immunoglobulin Index	1.9–3.1 (g/L)	Vehicle + AP	2.15 ± 0.92	3.5 ± 0	-
		HEBERSaVax	2.93 ± 1.54 a	2.67 ± 1.05 a	-
Alanine Aminotransferase (ALT)	1–56 (U/L)	Vehicle + AP	40.00 ± 50.91	30.00 ± 9.90	-
		HEBERSaVax	21.9 ± 13.31 a	25 ± 23.56 a	7.59 ± 3.79 b
Aspartate Aminotransferase (AST)	10.3–59.8 (U/L)	Vehicle + AP	58.00 ± 4.24	47.50 ± 14.85	-
		HEBERSaVax	59.2 ± 30.3 a	58.5 ± 28.11 a	47.15 ± 13.11 a
Alkaline Phosphatase (ALP)	60–850.4 (U/L)	Vehicle + AP	267.50 ± 279.31	291.00 ± 353.55	-
		HEBERSaVax	303.2 ± 230.47 a	264.2 ± 182.38 a	341.8 ± 266.66 a
Creatinine	0.48–1.1 (mg/dL)	Vehicle + AP	0.58 ± 0.02	0.05 ± 0.004	-
		HEBERSaVax	0.69 ± 0.14 a	0.21 ± 0.26 b	0.74 ± 0.145 a
Total Proteins	4.31–6.25 (g/dL)	Vehicle + AP	6.13 ± 0.18	4.93 ± 0.36	-
		HEBERSaVax	6.37 ± 0.5 a	5.65 ± 0.67 a	6.62 ± 0.48 a
Albumin	38.7–54.6 (g/L)	Vehicle + AP	41.10 ± 6.93	35.55 ± 2.05	-
		HEBERSaVax	44.67 ± 4.05 a	39.99 ±5.78 a	49.6 ± 4.99 b
Glucose	2.975–5.95 (mmol/L)	Vehicle + AP	4.49 ± 1.18	4.06 ± 0.68	-
		HEBERSaVax	3.44 ± 0.64 a	4.03 ± 1.26 a	3.68 ± 0.51 a
Cholesterol	2.09–5.17 (mmol/L)	Vehicle + AP	2.59 ± 0.08	1.52 ± 0.08	-
		HEBERSaVax	2.605 ± 0.42 a	1.97 ± 0.465 b	2.29 ± 0.7 a
Total Bilirubin	0–9.5 (mg/dL)	Vehicle + AP	2.75 ± 0.07	0.85 ± 0.92	-
		HEBERSaVax	3.59 ± 1.28 a	1.52 ± 0.59 b	2.44 ± 0.5 b
Triglycerides	0–1.01 (mmol/L)	Vehicle + AP	0.69 ± 0.21	0.42 ± 0.07	-
		HEBERSaVax	1.1 ± 0.41 a	0.36 ± 0.13 b	0.54 ± 0.25 a
Phosphorus	0.78–2.42 (mg/dL)	Vehicle + AP	1.39 ± 0.19	0.82 ± 0.35	-
		HEBERSaVax	1.64 ± 0.64 a	1.54 ± 0.22 a	1.48 ± 0.41 a
Urea	3.5–8.7 (mg/dL)	Vehicle + AP	6.29 ± 1.21	6.22 ± 1.60	-
		HEBERSaVax	7.37 ± 1.59 a	6.81 ± 1.33 a	6.61 ± 1.34 a
Calcium	1.89–2.89 (mmol/L)	Vehicle + AP	1.97 ± 0.13	1.79 ± 0.17	-
		HEBERSaVax	2.07 ± 0.09 b	1.89 ± 0.18 b	2.31 ± 0.09 a
Uric Acid	0–17 (mg/dL)	Vehicle + AP	0.50 ± 0.71	-	1.50 ± 2.12
		HEBERSaVax	1.2 ± 1.47 a	4.8 ± 1.36 b	0.3 ± 0.5 a
Gamma-Glutamyl Transferase (GGT)	1–199 (U/L)	Vehicle + AP	103.00 ± 69.30	108.50 ± 74.25	-
		HEBERSaVax	111.5 ± 63.95 a	92.1 ± 39.38 a	71.6 ± 37.92 b

Note: Complete blood counts and serum biochemistry were analyzed at baseline (Day 0), post-immunization (Day 56), and follow-up (Day 174). Data presented as mean ± SD (*n* = 10 per group). Each timepoint was compared to baseline (Day 0); different letters (a, b) indicate statistically significant differences between timepoints (*p* < 0.05)—paired *t*-test or one-way repeated measures ANOVA with Dunnett’s multiple comparisons test. “-” indicates unavailable samples. Not all biochemical parameters were assessed in the procedural control group at every time point due to sample volume limitations and a study design that focused on longitudinal analysis of the vaccinated cohort. Procedural control group data are presented where available.

## Data Availability

The data sets generated during the current study are available from the corresponding author on reasonable request.

## Supplementary Materials

The following supporting information can be downloaded at: https://www.mdpi.com/article/10.3390/vaccines14030230/s1, Figure S1. Antigen adsorption to Aluminum Phosphate and kinetics. Figure S2. Kinetics of anti-VEGF antibody titers in individual animals.

## Abbreviations

The following abbreviations are used in this manuscript:

AAALAC	American Association for the Accreditation of Laboratory Animal Care
ALT	Alanine transaminase enzyme
ANOVA	Analysis of Variance
AP	Aluminum Phosphate
AST	aspartate aminotransferase enzyme
CENPALAB	National Centre for Animal Breeding (Cuba)
CENTAURO	Name of the clinical trial series for HEBERSaVax
CFSE	Carboxyfluorescein Succinimidyl Ester (cell labeling dye)
CHO cells	Chinese Hamster Ovary cells (used in cytotoxicity assays)
CHO-rhVEGF	CHO cells expressing recombinant human VEGF ().
CICUAL	Committee for the Care and Use of Laboratory Animals (CIGB’s ethics board)
CIGB	Center for Genetic Engineering and Biotechnology (Havana, Cuba)
CTLs	Cytotoxic T Lymphocytes
ELISA	Enzyme-Linked Immunosorbent Assay
FDA	Food and Drug Administration
GMP	Good Manufacturing Practice
HEBERSaVax	Commercial name for CIGB-247 (the VEGF-based cancer vaccine)
HRP	Horseradish Peroxidase
IACUC	Institutional Animal Care and Use Committee
IgG	Immunoglobulin G
IMAC	Immobilized Metal Affinity Chromatography
ISO	International Organization for Standardization
NFAT-RE	Nuclear Factor of Activated T-cells Response Element (used in reporter assays)
NHP	Non-Human Primate
OECD	Organization for Economic Co-operation and Development
PBMCs	Peripheral Blood Mononuclear Cells
PBS	Phosphate-Buffered Saline
SD	Standard Deviation
SEM	Standard Error of the Mean
Th1/Th2	T-helper 1/T-helper 2 (immune response types)
TMB	3,3′,5,5′-Tetramethylbenzidine (ELISA substrate)
VEGF	Vascular Endothelial Growth Factor
VEGFR1/VEGFR2	VEGF Receptor 1/2
VSSP	Very Small Size Proteoliposomes (a bacterial-derived adjuvant)
