# Supplementary material for: Longitudinal Assessment of an 800 µg Dose of HEBERSaVax in Non-Human Primates over Six Months
Source: Vaccines (Basel). 2026 Feb 28;14(3):230. doi: 10.3390/vaccines14030230 (PMC13030131; doi:10.3390/vaccines14030230)
Supplement: Supplementary file 1 [file vaccines-14-00230-s001.zip › vaccines-4083250-supplementary.pdf]

Supplementary Figure S1

Characterization of the vaccine formulation revealed that adsorption to Al (OH)[3] was concentration-dependent, with incomplete adsorption observed at the highest antigen dose (800 μg), reaching ~75% after 1 hour (Supplementary Figure S1). This is likely due to saturation of the adjuvant's binding capacity at high antigen mass. While this may result in a portion of unabsorbed antigen in the vial, all inoculations were performed using a standardized protocol with a fixed adsorption time to ensure consistency. The absence of notable reactogenicity and the potent, adjuvant-typical immune response observed suggest that the formulation was effective. Future development and scale-up would include optimization of the adsorption process to maximize antigen binding across all dose levels.

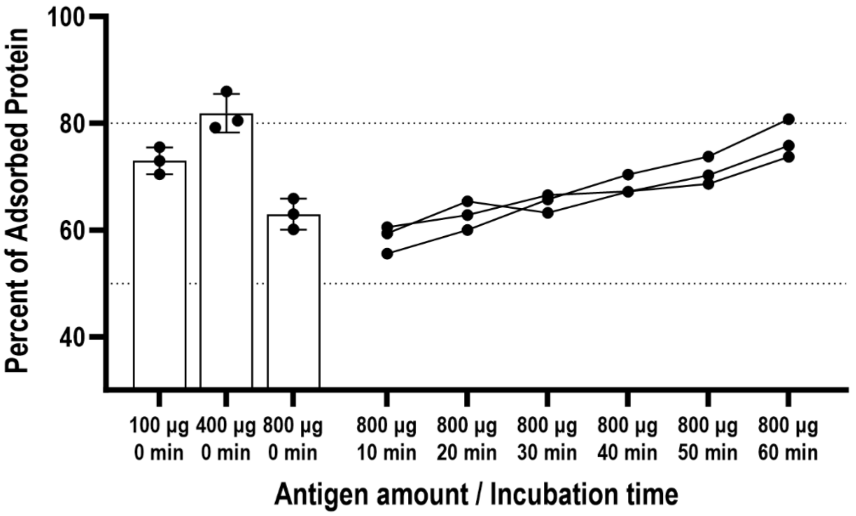

Figure S1. Antigen adsorption to Aluminum Phosphate and kinetics. The antigen-binding capacity of CIGB-247 to a clinical dose of aluminum phosphate ($AlPO_4$; 0.70 mg Al^{3+} per dose) was assessed by incubating increasing antigen concentrations (100–800 μg/mL) with the adjuvant in a 1:1 (v/v) ratio (1 mL antigen + 1 mL $AlPO_4$). Adsorption was evaluated immediately after mixing. Post-incubation, samples were centrifuged (4,000 rpm, 5 min), and free antigen was separated from adsorbed complexes by filtration through 0.2 μm Durapore membranes. Adsorption efficiency was quantified by analyzing 100 μL of non-adsorbed antigen using size-exclusion chromatography (Superdex 200 Increase XK10/30 column, 0.5 mL/min flow rate, 10 mM Tris pH 7.4 buffer). Results showed that 75–80% of the antigen adsorbed immediately after mixing at doses of 100 and 400 μg. At the highest dose (800 μg), initial adsorption reached ~60% (480 μg). For this concentration, adsorption kinetics were monitored

over 60 min at room temperature, with measurements taken at 10-min intervals. Adsorption increased gradually, reaching 75% after 1 hour. The dashed line indicates the acceptable adsorption range (50–80%).

Supplementary Figure S2

The analysis of antibody titers over time for each animal revealed a sustained and consistent humoral immune response, as shown by the mean and standard deviation of the entire cohort in Figure 1B. All animals displayed an initial induction phase, with peak titer levels achieved during the third and fourth immunizations. Following a rest phase marked by the expected decline in titers, a booster immunization on day 77 elevated antibody levels in all animals, demonstrating immune memory induction. This was followed by a final decline and stabilization phase (Supplementary Figure S2).

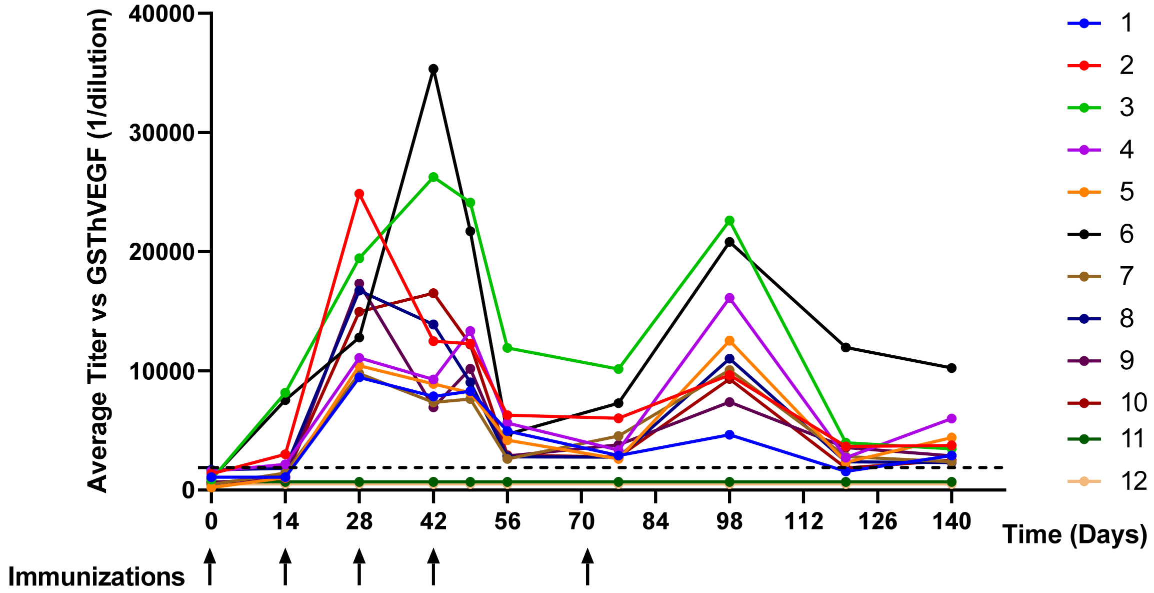

Figure S2. Kinetics of anti-VEGF antibody titers in individual animals. Titers of antibodies specific for human $VEGF_{121}$ were measured in serum by quantitative ELISA at different time points along the study. Data points represent anti-VEGF titer values from individual animals immunized with HEBERSaVax + AP (animals from 1 to 10) or Vehicle + AP (animals 11 and 12). The dashed line indicates the seropositivity threshold (3× pre-immune levels).
